# Advanced medical life support procedures in vitally compromised children by a helicopter emergency medical service

**DOI:** 10.1186/1471-227X-10-6

**Published:** 2010-03-08

**Authors:** Bastiaan M Gerritse, Annelies Schalkwijk, Ben J Pelzer, Gert J Scheffer, Jos M Draaisma

**Affiliations:** 1 Department of Anaesthesiology, Amphia Hospital, Breda, the Netherlands; 2 Department of Epidemiology, Biostatistics and Health Technology Assessment, Radboud University Nijmegen Medical Centre, Nijmegen, the Netherlands; 3 Department of Social Science Research Methodology, Radboud University Nijmegen Medical Centre, Nijmegen, the Netherlands; 4 Department of Anesthesiology, Pain and Palliative Medicine, Radboud University Nijmegen Medical Centre, Nijmegen, the Netherlands; 5 Department of Paediatrics, Radboud University Nijmegen Medical Centre, Nijmegen, the Netherlands

## Abstract

**Background:**

To determine the advanced life support procedures provided by an Emergency Medical Service (EMS) and a Helicopter Emergency Medical Service (HEMS) for vitally compromised children. Incidence and success rate of several procedures were studied, with a distinction made between procedures restricted to the HEMS-physician and procedures for which the HEMS is more experienced than the EMS.

**Methods:**

Prospective study of a consecutive group of children examined and treated by the HEMS of the eastern region of the Netherlands. Data regarding type of emergency, physiological parameters, NACA scores, treatment, and 24-hour survival were collected and subsequently analysed.

**Results:**

Of the 558 children examined and treated by the HEMS on scene, 79% had a NACA score of IV-VII. 65% of the children had one or more advanced life support procedures restricted to the HEMS and 78% of the children had one or more procedures for which the HEMS is more experienced than the EMS. The HEMS intubated 38% of all children, and 23% of the children intubated and ventilated by the EMS needed emergency correction because of potentially lethal complications. The HEMS provided the greater part of intraosseous access, as the EMS paramedics almost exclusively reserved this procedure for children in cardiopulmonary resuscitation. The EMS provided pain management only to children older than four years of age, but a larger group was in need of analgesia upon arrival of the HEMS, and was subsequently treated by the HEMS.

**Conclusions:**

The Helicopter Emergency Medical Service of the eastern region of the Netherlands brings essential medical expertise in the field not provided by the emergency medical service. The Emergency Medical Service does not provide a significant quantity of procedures obviously needed by the paediatric patient.

## Background

Advanced Life Support (ALS) for the pre-clinical management of vitally compromised children consists of endotracheal intubation and ventilation, intravenous or intra-osseous access with fluid replacement and administration of medication. The purpose of on-site advanced interventions is to stabilise the patient before transport to the hospital. These procedures are expected to reduce physiological deterioration, and thus to reduce mortality. However, this has never been proven on the basis of evidence. One of the confounding factors could be the (lack of) experience and the training required to perform the advanced interventions in a pre-clinical setting [1].

The Helicopter Emergency Medical Service (HEMS) was introduced in the Netherlands to provide optimal pre-clinical care for trauma patients by the Dutch government. The HEMS, consists of a physician (anaesthesiologist or trauma surgeon), a flight nurse and a pilot/driver. When the HEMS became operational, the Emergency Medical Service (EMS) frequently asked for assistance in stabilizing vitally compromised children. There were no paediatric HEMS data available in the Netherlands, research in other countries could not be easily extrapolated due to the international differences in HEMS and EMS organisations. However, there was a necessity to characterize the children involved to ameliorate HEMS and EMS care. The objective of this study was to evaluate the advanced medical interventions performed by the EMS and the HEMS in vitally compromised children, and to examine how often the HEMS provided additional medical care which was not or could not be provided by the EMS.

## Methods

Prospective cohort analysis of all HEMS calls for all paediatric emergencies for which the HEMS in the eastern part of the Netherlands (HEMS Netherlands-East) was called out, in the years 2001 to 2009. Only children under the age of 16 on the day of the emergency call were included. Approval from the ethical board of the Radboud University Nijmegen Medical Centre was obtained prior the onset of the study.

The HEMS Trauma Region Netherlands-East covers one of the four HEMS regions in the Netherlands, and covers an area of about 10,088 square kilometres in the eastern part of the Netherlands with 4.5 million inhabitants. Approximately 19.5% of the population in this area is under 16 years of age. The HEMS is called out either by the EMS dispatch centre (primary call) or by the EMS at the incident location (secondary call). The helicopter was active from January 2001 until September 2006 in daylight, and a physicians car was available during night and adverse weather. From September 2006 until today the helicopter crew is equipped with night vision goggles and fully operational 24 hours each day by helicopter. The physicians car is still available for foggy weather, and incidents close to the HEMS base (<10 kilometres).

HEMS physicians have received additional, extensive training (more than six months) in adult and paediatric emergency care, pain management and extrication techniques. HEMS physicians are authorised to perform advanced interventions that the paramedics of the Emergency Service (EMS) are not legally allowed to perform in the Netherlands. The paramedics of the EMS in the Netherlands are registered nurses with an additional training consisting of 175 hours of lectures concluded by exams. The EMS protocol in the Netherlands is a national protocol with precise description of procedures to follow. The paramedics of the EMS have only limited training and experience in vitally compromised children. However, the EMS-ambulance will be at the incident location in 15 minutes, due to the geographical distribution of EMS stations and time limits set by the government. The HEMS is called out according to a structured list of injury mechanisms or suspected morbidity. The HEMS can be cancelled before arrival if the vital signs of the patient are (almost) normal or if the patient has died. All medical procedures are applied in accordance with the appropriate advanced life support protocols (National EMS protocol for the EMS, guidelines of the Advanced Paediatric Life Support for the HEMS).

The registered data include age, sex, type of incident, physiological parameters (respiratory rate, heart rate, blood pressure, capnography), Glasgow Coma Scale (GCS), the pre-hospital treatment given, diagnosis in the emergency ward and survival until 24 hours after hospital admission. All patients examined by the HEMS were assessed according to the Munich modification of the NACA (National Advisory Committee for Aeronautics) score [2] (Table 1). The NACA score is a simple and both internationally and nationally established scoring system for grading disease and injury severity of patients in the preclinical setting. The worst clinical condition of the patient during pre-clinical management was the determining factor for classification, as described by the Munich modification of the NACA score [3]. It was also documented which of the pre-clinical advanced procedures were performed by the EMS or the HEMS. Advanced medical procedures were classified in three groups: procedures which are restricted to physicians under Dutch law (and thus restricted to the HEMS), procedures for which the HEMS is more experienced than the EMS and procedures for which the HEMS and EMS are equally experienced. This classification was created after a structured discussion between the HEMS and EMS management teams.

All data was recorded in an electronic patient data management system, custom made for the HEMS. The results were transferred into a data sheet (Excel™, Microsoft Seattle, USA), after which all data underwent statistical analysis and graphical depiction with SPSS Statistics 16.1™(SPSS Inc., Chicago, IL, USA). Pearson chi square was used for statistical comparisons, significance was defined as *p *< 0.05. Since the tables contain one or more cells with zero frequency, the exact significance of the obtained Chi square value was used instead of the asymptotic approximation.

**Table 1:** NACA Score

Score level	Patient status	Necessary intervention
I	Slight injury or illness	No medical intervention
II	Moderately heavy injury or illness	Ambulatory medical treatment
III	Heavy, but not life threatening injury or illness	Stationary medical treatment
IV	Heavy injury or illness, life threat cannot be excluded	Emergency medical measures
V	Acute mortal danger	Emergency medical measures
VI	Acute cardiac or respiratory arrest	Emergency resuscitation
VII	Death	

The National Advisory Committee for Aeronautics (NACA) developed a simple scoring system for patients receiving air transport during the Vietnam War.^2^

## Results

**Figure 1:**
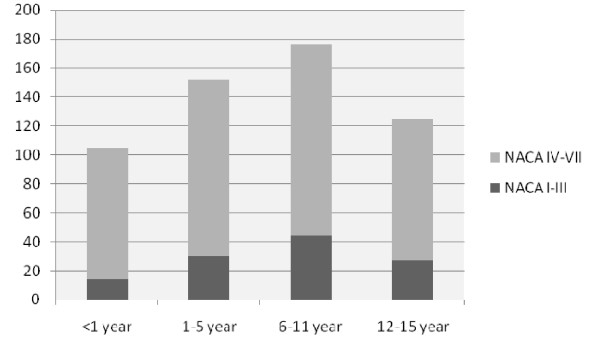
**Age-dependent distribution of NACA scores, differentiated according to numbers of infants (<1 year), toddlers (1-5 years), schoolchildren (6-11 years), adolescents (12-15 years)**. Pearson chi square p < 0.05

The HEMS had 803 calls involving children. In all cases the EMS was the first to arrive at the incident location. The average flight time of the HEMS was 9,6 minutes, ranging from 1 to 31 minutes. The time from HEMS alert to take-off of departure from the vehicle was an additional 2-5 minutes. Of these 803 calls, 245 (27%) were cancelled by the EMS before the arrival of the HEMS (199 children had normal physiological parameters, 27 children died and 19 calls other reasons). The HEMS examined and treated 558 children on scene with a mean age of 6.9 years (SD 5.3). Of these 558 children, 390 (70%) children had a trauma-related emergency and 168 (30%) children a non-trauma-related emergency. Of the children involved 115 (20.6%) had NACA scores of I-III, and 443 (79.4%) had NACA scores of IV-VII (medical cases 11% versus 89%, trauma cases 25% versus 75% respectively). (Pearson chi square p < 0.05). The youngest group of children (<1 year) had the relatively highest percentage of NACA scores IV to VII. (Figure 1).

Nine percent of all children were given cardiopulmonary resuscitation in the field (with a 24-hour survival rate of 26%). Ninety-five (17%) children died in the first 24 hours after the incident, of which 64 at the incident location. The emergency types with above-average mortality were all the non-trauma emergencies (except convulsions), near-drownings and burns. The emergency type 'congenital' includes all congenital disorders: cardiac, pulmonary or metabolic in a group of children with a wide variety of ages. The age range varied widely in the trauma related HEMS indications (Table 2). Of the 494 children who were transported from the incident location, 103 children (21%) were transported by helicopter. Children transported by ambulance without the HEMS physician had a significantly lower NACA score (Table 3).

**Table 2:** Paediatric HEMS incident according to initial EMS call

Initial HEMS call	Incidents n	Mean age (age range) Years	GCS (SD)	% 24-hour survival
1. preclinical childbirth/neonatal	29	0.1 (0-0.25)	7 (5)	79
2. congenital	14	4.9 (0.25-15)	4 (2.4)	29
3. infectious	27	2.0 (0.25-15)	6 (2.8)	67
4. convulsions	18	3.4 (0.4-15)	7 (3.8)	94
5. asphyxia	35	5.5 (0.1-14)	10 (5.0)	71
6. CPR general (non-neonatal)	45	4.9 (0.1-15)	5 (5.7)	49
7. Near-drowning	40	4.3 (0.6-15)	7 (3.8)	80
8. Burns	12	4.5 (0.2-11)	13 (4.6)	50
9. Pedestrian versus motor vehicle	60	8.1 (0.2-15)	9 (5.0)	85
10. Cyclist versus motor vehicle	67	11.3 (0.3-15)	8 (4.7)	90
11. Passenger in motor vehicle	88	8.2 (0.3-15)	12 (4.9)	91
12. Moped	30	13.3 (1-15)	11.2 (5.3)	97
13. Fall	55	6.7 (0.3-15)	11.7 (4.7)	95
14. Equestrian	14	10.7 (4-15)	7.9 (5.2)	100
15. Other	24	7.5 (0.4-15)	12 (5.4)	92
				
Total	558	6.9 (0-15)	8.9 (5.0)	83

**Table 3:** Transportation of patients

	n	**NACA I-III**^**@**^	**NACA IV-VII**^**@**^
No transportation, dead on scene	64	0	64
Ambulance, with HEMS physician	273	20	253
Ambulance, without HEMS physician	118	95	23
Helicopter transport because of distance to hospital	25	0	25
Helicopter transport because of condition of patient	76	0	76
Interhospital transfer	2	0	2

^@^NACA groups: Pearson chi square p < 0.05

A total of 1649 advanced medical procedures were provided by the HEMS to the 558 children, an average of 3.0 procedures per child (table 4). Advanced medical procedures (n = 818) restricted to the HEMS were given to 65% (n = 365) of the children. Medical procedures (n = 831) for which the HEMS is more experienced than the EMS were provided to 78% (n = 438) of the children (Table 4). In 482 children (86%) a medical procedure from one or both of these groups was performed by the HEMS.

**Table 4:** Pre-hospital medical procedures

Restricted to HEMS		HEMS more experienced	HEMS	EMS
	n		n	n
Hypnotics*	147	Unsuccessful endotracheal intubation^®^	0	20
Muscle relaxants#	146	Successful endotracheal intubation^®^	214	66
Chest tube	5	Peripheral venous canula	272	304
Central venous line	12	Intraosseous access	68	31
Hypertonic fluid&	104	Intraosseous access and CPR	19	27
Antibiotics∀	26	Pain management**	149	45
Physician transfer	376	Medication for ALS⊥	109	28
Venous cutdown	2			
Total	818		831	521

*Hypnomidate, midazolam, propofol, s-ketamine (hypnotic dose)

#Suxamethonium, rocuronium

&Mannitol, hyperhaes

∀ Cefazolin, ceftriaxon

**Fentanyl, Alfentanyl, locoregional anaesthesia, s-ketamine (analgetic dose)

⊥ Amiodarone, atropine, dobutamine, epinephrine

^® ^Successful versus unsuccessful endotracheal intubation: Pearson chi square p < 0.05

A medical procedure in which the HEMS is more experienced than the EMS is endotracheal intubation. EMS paramedics arriving at the incident location before the arrival of the HEMS intubated 86 children, with a success rate of 77% (n = 66). A part of these children have been further described in a previous publication by these authors [4]. In twenty of these 86 children an emergency correction of the endotracheal tube or ventilator settings was performed by the HEMS upon arrival: oesophageal intubation (n = 13), inappropriately sized endotracheal tube without cuff making positive pressure ventilation impossible (n = 5) and potentially lethal ventilator settings (n = 2) (>300% of recommended ventilator settings). The HEMS intubated 214 children with 100% success. Successful intubation was defined as symmetrical breath sounds by auscultation, and a positive mainstream capnography, followed by mechanical ventilation with normal airway pressures. These measures only partially eliminate the presence of bronchial intubation, but would make it more rare. An acknowledged and corrected primary esophageal intubation by HEMS was registered as a success. Oxygen saturation was often difficult to register during the medical intervention, and the fall of saturation was not registered during the endotracheal intubation. In cardiopulmonary resuscitation without any capnography reading, the endotracheal intubation was confirmed by repeat laryngoscopy. The difference in the number of successful endotracheal intubations by the EMS and the HEMS is significant (Chi square p < 0.05). Twelve percent (n = 39) of the children with a GCS > 7 were intubated by the HEMS (compromised airway, pain management or to facilitate transportation by helicopter).

Intraosseous access was obtained in 99 children, 68 by the HEMS and 31 by the EMS. Eighty-seven percent (n = 27) of all children provided with intraosseous access by the EMS were in cardiopulmonary arrest, versus 28% (n = 19) in the HEMS group.

Pain management was given to 35% (194/558) of the children. The medication of choice was fentanyl or alfentanyl, occasionally lidocaine for infiltration anaesthesia and levobupivacaine for peripheral nerve blocks. The youngest child provided with pain management by an EMS paramedic was four years old; by the HEMS two months old. No detrimental effects of the pre-clinical application of analgesics were recorded.

## Discussion

There are no studies that show convincingly that a physician-based EMS leads to a decrease in overall mortality or morbidity of pre-clinically treated patients [5]. However, in those patients requiring advanced airway management or other invasive procedures, as well as fluid management and pharmacotherapy, adding a specialist physician to the pre-hospital emergency care can increase survival and improve outcome [5].

The children in this study who were examined and treated by the HEMS constitute a particularly compromised group. Nine percent of all children were given cardiopulmonary resuscitation in the field (with a 24-hour survival rate of 26%). Eich described 2271 paediatric emergencies in a comparable study on EMS and HEMS in Germany [6]. In this study, 72.7% of the children had a NACA score of I-III and 27.3% had a NACA score of IV-VII (versus 20.6% and 79.4% respectively in our study). (Pearson chi square p < 0.05). This discrepancy may be caused by profound differences between the Netherlands and Germany in the pre-clinical emergency care for vitally compromised children, due to differences in infrastructure, dispatching protocols, geography or training of EMS. Still, the conclusions stated in the study of Eich are even more valid to the HEMS in the Netherlands. The HEMS in our study encounters a high incidence of paediatric emergencies in children, therefore "...skills in paediatric airway management, cardiopulmonary resuscitation and intraosseous canulation in all age groups are essential..." [6].

The youngest patients have the highest NACA scores. Certain causes of a preclinical vital threat occur only in early childhood, like unexpected childbirth and duct-dependent congenital heart disease. Other causes of life-threatening events, like sepsis, convulsions and near-drowning, occur especially in toddlers and younger children [6]. These life-threatening events have a low rate of survival in this study. As advanced life support procedures are considered to be more difficult in younger children, special training in these cases should be provided for optimal performance of the HEMS. As shown in the age range variation in table 2, young children can be involved in any kind of trauma incident.

Zautcke e.a. studied the amount of skill deterioration in 40 paramedics after graduation [7]. Examination consisted of the practical aspects of airway management, spinal immobilization and intravenous fluid therapy in relation to their final school examination. As a group, the study scores were significantly lower than the graduation scores except in spinal immobilization and extremity immobilization. A continuing education and recertification process is necessary to identify and correct deficiencies in performance. The number of 20 failed intubations or lethal ventilator settings is unacceptably high. The rate of failed endotracheal intubations by the EMS-paramedics has relatively diminished in the last years of this study in comparison to our previous publication on this subject [4]. The reasons for this trend are unknown, still any not-recognised oesophageal intubation can have catastrophic consequences.

It has been clearly shown that experience is crucial for successful preclinical endotracheal intubation [8,9]. A far better option for the paramedics in the EMS would be the maintenance of oxygenation by bag-valve-mask ventilation until the arrival of an HEMS or arrival in the emergency ward [4,10,11]. Theoretically, there are clear advantages to preclinical endotracheal intubation: facilitation of artificial ventilation, protection against aspiration, facilitation of transport by helicopter. This should, however, never compromise the application of supplemental oxygen and adequate ventilation.

Intraosseous access is recommended in vitally compromised children if intravenous access is difficult or impossible, and can also be effective in adults. As intraosseous access by EMS-paramedics is predominantly used in children in cardiopulmonary arrest, a potentially large group of vitally compromised children were left without this useful device. The HEMS in this study did provide intraosseous access to children outside the CPR group. Although the EMS paramedics are trained in intraosseous access, it is not widely applied: only 31% of all intraosseous access was provided by the EMS paramedics. The infrequent use of intraosseous infusion compared to other advanced life support skills in hospital and by paramedics and HEMS has been described [12,13]. Still, several studies have shown that the placement of an intraosseous line is easy, fast and has a high success rate [14-16].

The number of children who needed pain medication but did not receive it from the EMS is high: 77%. No child under the age of four years (e.g. the burn victims) received any pain medication from the EMS. The safe delivery of adequate analgesia is a priority in pre-hospital care; ketamine is relatively safe when used by physicians [17]. In a review by Thomas, clear evidence supporting the safety of pre-hospital analgesia was provided. Pain relief can be improved in an EMS or HEMS by balancing the desire to do no harm, and the unacceptable fact of allowing needless suffering [18]. This clearly calls for additional education and standards to improve pre-clinical pain management. The potential fear of the EMS of causing ventilatory depression has to be addressed.

There are several limitations to this study. Due to the nature of the health care provided, a blind prospective study was not feasible. The added value of adding a HEMS to the EMS was quantified by the number of medical procedures, with special attention for the procedures for which the EMS is neither certified nor experienced. There was no follow-up after 24 hours of admission, so actual survival until hospital discharge was unknown. The reason for this was the transportation of patients to hospitals out of the primary HEMS region.

## Conclusion

The HEMS of the eastern part of the Netherlands provides essential additional medical expertise not provided by the EMS. The only formal paediatric indication for HEMS at this moment is the paediatric cardiopulmonary resuscitation. This study calls for a lower threshold for HEMS activation in any serious incident involving children, preferably based on the type of primary emergency call.

Sixty-five percent of the vitally compromised children received a preclinical medical procedure restricted to a physician, 78% received a medical procedure for which a physician was more experienced. The majority of all patients encountered by the HEMS had a NACA score of IV-VII. As the younger patients had a higher NACA score, special attention should be given to training and the provision of advanced life support procedures for younger children.

Successful endotracheal intubation and subsequent appropriate ventilation in children is a difficult task for EMS paramedics; preclinical endotracheal intubation of children calls for an experienced physician. The use of intraosseous access devices and the use of analgesics by EMS paramedics could be improved. Further investigation into the pre-hospital care for vitally compromised children is necessary.

## Key Messages

• The HEMS of the eastern part of the Netherlands provides essential additional medical expertise not provided by the EMS.

• The majority of all patients encountered by the HEMS had a NACA score of IV-VII.

• A substantial proportion of all endotracheal intubations by EMS paramedics resulted in potentially lethal complications.

• The use of intraosseous access devices and the application of analgetics in the field can be improved.

## Abbreviations

EMS: Emergency Medical Service; HEMS: Helicopter Emergency Medical Service; NACA: National Advisory Committee for Aeronautics; SPSS: Statistical Package for the Social Sciences; ALS: Advanced life support; GCS: Glasgow Coma Scale; CPR: Cardiopulmonary resuscitation; SD: Standard deviation.

## Competing interests

The authors declare that they have no competing interests.

## Authors' contributions

BMG: main author, design of study. AS: data acquisition, design of research data base. BJP: statistical analysis, scientific structure. GJS: expert in anaesthesiology and critical care, medical reviewer. JMD: expert in emergency paediatric care, medical reviewer. All authors read and approved the final manuscript.

## Pre-publication history

The pre-publication history for this paper can be accessed here:

http://www.biomedcentral.com/1471-227X/10/6/prepub

## References

[B1] Ummenhofer W Scheidegger D Role of the physician in prehospital management of trauma: European perspective Current Opinion in Critical Care 2002 8 559 65 10.1097/00075198-200212000-00013 12454542

[B2] Veldman A Fischer D Brand J Racky S Klug P Diefenbach M Proposal for a new scoring system in international interhospital air transport J Travel Med 2001 8 154 157 (Table 1, page 154) 11468122 10.2310/7060.2001.24467

[B3] Lackner CK Schlechtriemen T Burghofer K Stolpe E Altemeyer KH The Munich NACA score: Modification of the NACA score for preclinical emergency medicine Notfall und Rettungsmedizin 2005 8 2 109 111 10.1007/s10049-005-0719-5

[B4] Gerritse BM Draaisma JMT Schalkwijk A van Grunsven PM Scheffer GJ Should EMS-paramedics perform paediatric tracheal intubation in the field? Resuscitation 2008 79 225 229 10.1016/j.resuscitation.2008.05.016 18684547

[B5] Timmermann A Russo SG Hollmann MW Paramedic versus emergency physician emergency medical service: role of the anaesthesiologist and the European versus the Anglo-American concept Curr Opin Anaesthesiol 2008 21 222 227 10.1097/ACO.0b013e3282f5f4f7 18443493

[B6] Eich C Russo SG Heuer JF Timmermann A Gentkow U Quintel M Roessler M Characteristics of out-of-hospital paediatric emergencies attended by ambulance- and helicopter-based emergency physicians Resuscitation 2009 80 888 892 10.1016/j.resuscitation.2009.05.008 19520484

[B7] Zautcke JL Lee RW Ethington NA Paramedic skill decay Journ Emerg Med 1987 5 505 512 10.1016/0736-4679(87)90214-9 3429822

[B8] Boswell WC McElveen N Sharp M Boyd CR Frantz EI Analysis of prehospital pediatric and adult intubation Air Med J 1995 14 125 128 10.1016/1067-991X(95)90513-8 10151151

[B9] Pepe PE Copass MK Joyce H Prehospital endotracheal intubation: rationale for training emergency medical personnel Ann Emergency Medicine 1985 14 11 1085 1092 10.1016/S0196-0644(85)80927-6 3931512

[B10] Cooper A DiScala C Foltin G Tunik M Markenson D Welborn C Prehospital endotracheal intubation for severe head injury in children: a reappraisal Sem in Pediatric Surgery 2001 10 1 3 6 10.1053/spsu.2001.19379 11172563

[B11] Gausche M Lewis RJ Stratton SJ Haynes BE Gunter CS Goodrich SM Poore PD McCollough MD Henderson DP Pratt FD Seidel JS Effect of Out-of-hospital pediatric endotracheal intubation on survival and neurological outcome JAMA 2000 283 783 790 10.1001/jama.283.6.783 10683058

[B12] Glaeser PW Helmich TR Szewczuga D Losek JD Smith DS Five-year experience in prehospital intraosseous infusions in children and adults Ann Emerg Med 1993 22 1119 24 10.1016/S0196-0644(05)80975-8 8517560

[B13] Helm M Hauke J Bippus N Lampl L Die intraossäre Punction in der präklinischen Notfallmedicin Der Anaesthesist 2007 56 18 24 10.1007/s00101-006-1124-2 17195071

[B14] Curran A Sen A Best evidence topic report. Bone injection gun placement of intraosseous needles Emerg Med J 2005 22 5 366 Review. 10.1136/emj.2005.024406 15843711 PMC1726784

[B15] Calkins MD Fitzgerald GF Bentley TB Burris D Intraosseous infusion devices: a comparison for potential use in special operations J Trauma 2000 48 1068 74 10.1097/00005373-200006000-00012 10866253

[B16] Gerritse BM Scheffer GJ Draaisma JMT Prehospital intraosseous access with the bone injection gun by a Helicopter Emergency Medical Service J Trauma 2009 66 1739 41 10.1097/TA.0b013e3181a3930b 19509638

[B17] Bredmose PP Lockey DJ Grier G Watts B Davies G Pre-hospital use of ketamine for analgesia and procedural sedation Emerg Med J 2009 26 62 4 10.1136/emj.2007.052753 19104109

[B18] Thomas SH Shewakramani S Prehospital trauma analgesia Journal of Emerg Med 2008 35 45 57 10.1016/j.jemermed.2007.05.041 17997072

